# Adversarial Learning with Bidirectional Attention for Visual Question Answering

**DOI:** 10.3390/s21217164

**Published:** 2021-10-28

**Authors:** Qifeng Li, Xinyi Tang, Yi Jian

**Affiliations:** 1 Shanghai Institute of Technical Physics of the Chinese Academy of Sciences, Shanghai 200083, China; gq227@mail.sitp.ac.cn (X.T.); jianyi@mail.sitp.ac.cn (Y.J.); 2 School of Electronic, Electrical and Communication Engineering, University of Chinese Academy of Sciences, Beijing 100049, China; 3 Key Laboratory of Infrared System Detection and Imaging Technology, Chinese Academy of Sciences, Shanghai 200083, China

**Keywords:** bidirectional attention, adversarial learning, visual question answering, attention visualization, feature fusion, feature selection, attention mechanism

## Abstract

In this paper, we provide external image features and use the internal attention mechanism to solve the VQA problem given a dataset of textual questions and related images. Most previous models for VQA use a pair of images and questions as input. In addition, the model adopts a question-oriented attention mechanism to extract the features of the entire image and then perform feature fusion. However, the shortcoming of these models is that they cannot effectively eliminate the irrelevant features of the image. In addition, the problem-oriented attention mechanism lacks in the mining of image features, which will bring in redundant image features. In this paper, we propose a VQA model based on adversarial learning and bidirectional attention. We exploit external image features that are not related to the question to form an adversarial mechanism to boost the accuracy of the model. Target detection is performed on the image—that is, the image-oriented attention mechanism. The bidirectional attention mechanism is conducive to promoting model attention and eliminating interference. Experimental results are evaluated on benchmark datasets, and our model performs better than other models based on attention methods. In addition, the qualitative results show the attention maps on the images and leads to predicting correct answers.

## 1. Introduction

Computer vision and natural language processing have developed rapidly for a long time. As a result, more and more cross fields of machine vision and natural language processing have emerged. Visual question answering (VQA) is one of the most important research fields. To better solve the problem of visual question answering, it is necessary to understand the image that the method focuses on and the deep visual features contained in the correct area of the image.

The attention mechanism is a human-specific brain signal processing mechanism [1,2,3,4]. In a world where we unintentionally or intentionally receive different signals every day, mainly from sight and hearing, top–down attention mechanisms allow us to quickly notice important signals in the environment. Although this kind of attention pays attention to the important signals of the surrounding environment quickly, this kind of unidirectional attention is still insufficient in efficiency. That is, there is no in-depth mining of features from the perspective of images. So, we choose a bidirectional attention method, including bottom–up attention and top–down attention, to make up for this shortcoming [5].

The answer of a good VQA model should be no different from that of humans. The adversarial learning is motivated by Generative Adversarial Nets (GANs). In the process of recognizing objects, humans enhance the cognitive ability of objects through contrast. Since the adversarial learning was proposed, it has achieved a series of good achievements in the field of artificial intelligence [6,7,8]. In this article, we propose using deep learning features with two different pictures, which match with related questions to infer the best answer. Through adversarial learning, the model will pay attention to significant parts of the target image that are distinguished from the adversarial image [9].

**Figure 1:**
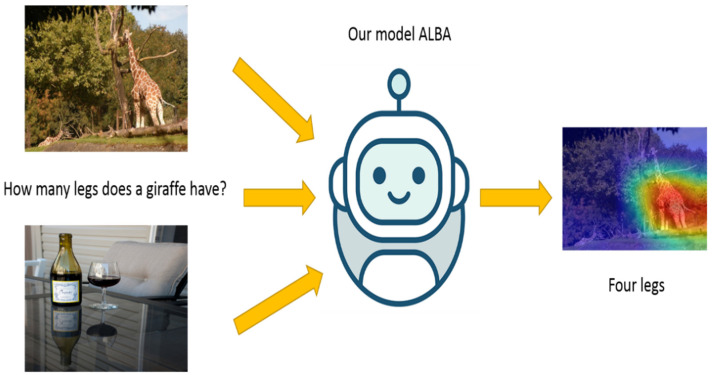
As an example, our model fuses features of the target image and the adversarial image according to the question, predicts the correct answer, and visualizes the attention.

In this paper, we propose a bidirectional attention mechanism through adversarial learning for improving the understanding facility for the fusion of language and visual representations. The main flow of the model followed is illustrated in Figure 1. Given a question, a related image, and an unrelated image, we use a bidirectional attention network to obtain a target attention embedding, which can find the part of the image that is most relevant to the problem. Meanwhile, by subtracting the target attention embedding and the adversarial attention embedding, we conduct the adversarial learning to filter out useless parts of the image and highlight the most meaningful image part for the question.

## 2. Related Work

In this section, we briefly introduce the previous research of VQA, especially the development in the field of adversarial learning and attention mechanism.

### 2.1. Attention Mechanism

The attention mechanism has proved to be very effective in many tasks, and visual question answering is no exception. Several deep neural networks based on an attention mechanism have been proposed for visual question answering, in which question-oriented attention combined with image regions are commonly used.

The creation and use of attention maps have aroused great interest. Lu et al. [10] proposed a co-attention model for visual question answering that figures out image and question attention. Shih at al. [11] presented a way of learning to answer visual questions by selecting image areas related to text-based queries. Yang et al. [12] created a stacked attention network, which generates multiple attention maps on the image in a sequential manner, in order to increase the reasoning steps and improve the reasoning ability.

Early VQA research started with the attention mechanism concerning the question. Xu et al. [13] proposed a spatial memory network to apply question-guided spatial attention on certain parts of the image, which are related to either a single word or the whole question. Lu et al. produced the co-attention mechanism that creates attention on a certain area of the image and related question words. Shi et al. [14] suggested Question Type Guided Attention (QTA), which takes advantage of information about the question type to efficiently balance the bottom–up and top–down visual features.

### 2.2. Glove

In the training or testing phase, the input of each instance is a target image, an adversarial image, and a question. Spaces and punctuation are used to divide the question into individual words. Any number is taken as a word. The maximum number of words in each question is reduced to 15 words, which can keep the semantics of the question and computational efficiency. Extra words are automatically omitted. Probably only 0.1% of the questions exceed 15 words. Each word is encoded by the glove embedding algorithm [15], which is a look-up table with 300-dimensional vectors. The public algorithm we used has been trained on Wikipedia. Questions less than 15 words are padded zero at the end of the question. The size of a resulting vector matrix is 15 × 300, and then, it is passed through the GRU (Gated Recurrent Unit). After processing fifteen words sequentially, we got the 512-dimensional question vectors.

### 2.3. Faster-RCNN

The same limitation of most models mentioned above is to use a full image to create global features to represent the visual input. This will feed noise to the model and then negatively affect the final result. In fact, we make use of Faster-RCNN as a segmentation method to divide the image into 36 different regions which are 36 different pooled convolutional feature vectors [16,17]. In this way, we can obtain image features directly related to the question, eliminate noise interference, and facilitate the attention of the model to the most relevant objects.

### 2.4. Adversarial Learning

The method of adversarial learning is inspired by human perception mechanisms [18]. In the process of answering questions, the more suitable and reasonable answers are given out by people comparing the differences between the information to find contradictions and differences [19,20,21,22]. Adversarial learning has made a series of good achievements in the field of artificial intelligence. Chen et al. [23] take adversarial learning as a segmentation network to automatically create realistic composite images. Wu et al. [24] presented an approach of combining Reinforcement Learning and Generative Adversarial Networks whose advantage is to overcome the relative shortage of training data. Our work is motivated by the effectiveness of the adversarial learning, but we cautiously extend it to our application VQA. Through the adversarial network, the model will pay attention to two parts: one is the area that is most relevant to the question in the target image, and the other is the part of the adversarial image that is the most different from the question. By contrast between two images, the mode learned how to find the best answer.

## 3. Proposed Method

This section is divided into five subheadings, which introduce the internal structure of the model, the experimental methods, and the experimental procedures in detail.

**Figure 2:**
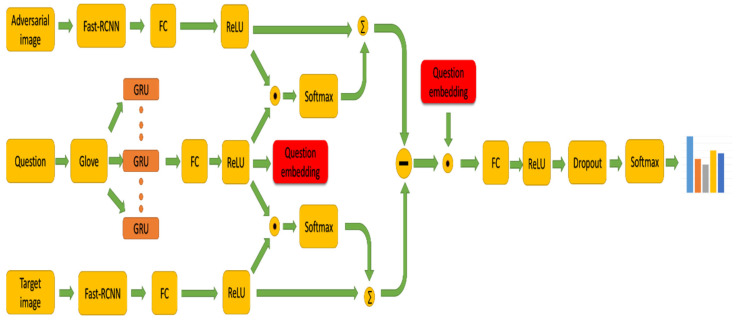
Overview of the proposed model. The target image–question pair and the adversarial image–question pair perform feature extraction and feature fusion operations respectively and then predict the correct answer through the classification network.

In this section, we introduce the architecture of our model in Figure 2. As the Figure 2 shows, it demonstrates the combination of adversarial learning and bidirectional attention mechanism to solve the problem of visual question answering, with bidirectional attentions over regions of the image. In order to maintain transparency, we will list the exact steps and specific hyperparameter values in the model in detail, which result in its best performance.

### 3.1. Finding Adversarial Images

Although few images in the VQA2.0 dataset are somewhat similar, it is necessary to choose an image that is very different from the target image in order to ensure the effectiveness of the adversarial learning network.

In our experiment, we use a histogram to compare the similarity of two images and select adversarial images. The histogram correlation d is given by [25,26]:

(1) $$d\left(H_{1},H_{2}\right)=\frac{\sum_{I}\left(H_{1}\left(I\right)\;-\;{\bar{H}}_{1}\right)\left(H_{2}\left(I\right)\;-\;{\bar{H}}_{2}\right)}{\sqrt{\sum_{I}{\left(H_{1}\left(I\right)\;-\;{\bar{H}}_{1}\right)}^{2}\sum_{I}{\left(H_{2}\left(I\right)\;-\;{\bar{H}}_{2}\right)}^{2}}}$$

(2) $${\bar{H}}_{k}=\frac{1}{N}\sum_{J}H_{k}.$$

*N* is the total number of histogram bins. H_1_ and H_2_ represent the histogram of the target picture and the histogram of the confrontation picture respectively. ${\bar{H}}_{k}\;$ denotes the average of the histogram.

First, we select the target image as the reference image. Second, we randomly pick up another image in the dataset. Third, we convert two images into HSV format. Finally, we calculate the correlation of two image histograms. The greater the correlation of the histogram, the more similar the two images. If the correlation between the two pictures is less than 0.2, we select these two images as a set of input.

### 3.2. Image Feature Selection

As a target detection method based on convolutional neural network, Faster-RCNN first uses a set of basic layers including convolutional layers, ReLU layers, and pooling layers to extract the feature maps of the image [17]. The feature maps are shared for the subsequent RPN layer and fully connected layer.

In the RPN network, in order to achieve the matching of the common feature box in the original image with the candidate box of each target [27], it is necessary to use anchor boxes to set the target block diagram. Each feature vector in the convolution feature map corresponds to a small area in the original image, and the size of the area is determined by the convolution kernel. With each point on the feature map as the center, anchor boxes of different sizes and different proportions can be generated, corresponding to different areas on the original image. As a result of the actual changes in the size of the anchor boxes and the size of the target, usually the corresponding area cannot be completely matched with the target frame. This results in the absence of the target in the RPN built-in candidate area. In order to obtain a more accurate candidate area and complete the area proposal link, it is necessary to optimize the preset area and generate k frames of different proportions and different sizes to adapt to the target with each anchor point as the center.

The pooling layer extracts proposal feature maps after integrating feature maps and proposals information and sends them to the subsequent fully connected layer to determine the target category.

The classification layer uses proposal feature maps to distinguish the category of the proposal and utilizes bounding box regression to obtain the final precise position of the detection frame [28].

### 3.3. Bidirectional Attention

The target image is passed through a Faster R-CNN to obtain k × 2048-dimensional vector representation, where K represents the number of features on an image. Each feature is a 2048-dimensional vector, which encodes a specific area on the image. The Faster R-CNN can extract K (K = 36) distinctive objects from the image, which greatly provides the information related to the problem. 

Our model adopts the attention method corresponding to the human question-guided model, with different features from the model of Anderson et al. [5] that focuses on the attention mechanism of image features.

The output of the Faster R-CNN (v_i_) first passes through a Rectified Linear Unit (ReLU) activation layer [29] to avoid gradient explosion and gradient disappearance, and then, it passes through a fully connected layer to get a K × 512- dimensional vector m_i_. w_i_ is a learned parameter vector

m_i_ = w_i_f(v_i_). (3)

Each feature vector mi (i = 1…K) and question embedding q are combined with an element-wise multiplication [30]. The resulting vector g is a fusion of image features and embedding of the question.

g = m_i_ ⊙ q (4)

The fusion vector is normalized over all features with a softmax function.

λ = softmax(g) (5)

Then, the image features m_i_ (i = 1…K) from an entire image are weighted by the normalized values and summed to obtain a 512-dimensional vector V_T_.

(6) $$V_{T}=\sum_{i=1}^{K}\lambda_{i}m_{i}$$

The same processing steps are taken for the adversarial image to get a 512-dimensional vector V_A_. Note that this attention mechanism is two-way attention, as opposed to simple one-way attention and a more complicated attention mechanism.

### 3.4. Adversarial Attention

Based on the adversarial learning, some features of the representation of the target image V_T_ not related to the question have been removed by subtracting the image features of the adversarial image V_A_. So, we get some image features without redundancy. The representations of the target image V_T_ and of the adversarial image V_A_ are processed by element-wise subtraction. W_1_ is a learned parameter vector.

h = w_1_(V_T_ ㊀ V_A_) (7)

### 3.5. Classifier

In the train set, each question has 10 answers, and these answers constitute the label of training classification, in which we select the correct answer appearing more than 7 times. Therefore, we got a total of N = 3129 candidate answers. Improving the efficiency of the VQA model is actually improving the accuracy of the multi-label classification task. In the VQA2.0 training set, each answer is labeled with soft accuracies in the range of 0 to 1. The score of each answer is different based on the judgment of different people.

The fusion of image features and question embedding h is fed into a ReLU activation layer, passes through a fully connected layer and a dropout layer, and then finally passes through a softmax layer [31] to predict a score for each candidate answer.

(8) $${\hat{s}}_{ij}=\mathrm{softmax}\;\left\{f_{d}\left[w_{s}.\;f_{s}\left(h\right)\right]\right\}$$

where w_s_ ∈ R^N × 512^ is a learned weight matrix, f_s_ is a ReLU activation layer, and f_d_ is a dropout layer (*p* = 0.5).

The softmax layer normalizes the answer score to (0,1). Then, we use a loss method similar to binary cross-entropy [32]. This layer can be seen as using logistic regression to predict the correct answer. The final function is

(9) $$L=-\sum_{i}^{M}\sum_{i}^{M}s_{ij}\log\left({\hat{s}}_{ij}\right)-\left(1-s_{ij}\right)\log\left(1-{\hat{s}}_{ij}\right)$$

where M and N represent respectively the number of training questions and the number of candidate answers. S is the soft ground-truth scores of truth answers. Soft scores as targets maintain more effective target signals than binary labels.

## 4. Evaluation

In the section, for analyzing our proposed VQA model, we focus on analyzing how the glove algorithm affects the question embedding and accuracy of the model performance. In addition, we present additional experiments to prove the superiority of our model by comparing with other models. Finally, we analyze the specific impact of the bidirectional and adversarial attention mechanism.

### 4.1. Dataset

The VQA2.0 dataset [33] is the richest and most extensive dataset, which is the updated version of VQA1.0 dataset. It increases the diversity of answers to each question to minimize the impact of dataset priors. The answers to each question are divided into three categories: yes/no, number, other. Each question includes ten candidate answers. The VQA2.0 dataset was selected as the official dataset of the VQA Challenge, which includes 443,757 train, 214,354 validation, 447,793 test questions.

### 4.2. Experimental Setting

Our model adopts single network learning instead of ensemble learning. Each of our networks is trained based on the VQA2.0 dataset. The model is trained multiple times on the VQA2.0 training dataset, and the optimal parameters are selected. The highest accuracy rate on the validation test VQA2.0 is selected as the result. We performed each step of the experiment four times, each time using different random seeds. In order to evaluate the performance of the model, the standard VQA metric [34] is used to calculate the accuracy, which minimizes the accidental noise from the annotators of the ground truth answers.

### 4.3. Ablation Study

In order to obtain the optimal model, we have done relevant comparative experiments through the controlled variable method. By changing the model parameters and model structure, the most suitable architecture and hyperparameter values can be selected. In addition, we evaluate the sensitivity of each part to the final experimental results.

Image similarity is an important variable that affects the model. Our model uses adversarial images with similarity less than 0.2, and the control group uses adversarial images with similarity greater than 0.2. Compared with the control group, it can be seen from the accuracy rate that almost every item has been improved by almost 1%. This means that the smaller the similarity between two images, the higher the accuracy of the model.

Glove word embedding of dimension 300 followed by a one-layer GRU is adopted in our reference model. Three other word-encoding methods are selected to compare with our reference model. The performance of the first and third methods is slightly reduced by about 1%. The second method is the worst of the three.

Our image features are obtained through a combination of a Faster R-CNN framework and 101-layer ResNet. A fixed threshold is used to limit the number of object detections, and the number of features K perfectly matches the content of the image. The range of parameters K is from 0 to 100. In our experiment, we take K = 36 as the parameter. In this way, we can lower computation and reduce complexity. A 200-layer ResNet as a main option is used in our comparative experiment. The performance of the ResNet-200 features downsampled to 7 × 7 (K = 49) dramatically drops to 69.59%. The ResNet-200 global features (K = 1) are expectedly even worse.

It can be seen from the experimental data on Table 1 that adopting adversarial learning helps improve the accuracy and robustness of the model. Obviously, the model is not only sensitive to the correct answer but can also effectively identify the wrong answer.

### 4.4. Comparison with Existing Methods

In the section, we will introduce the effectiveness and functions of our proposed network. In order to ensure the fairness of the comparison process, the published model we chose does not use additional datasets for training. Table 2 shows the comparison of the results between the proposed method and the other published methods on VQA2.0. Obviously, our model outperforms performs better than the published models in the table by a margin of 0.6% on the test-standard and test-dev dataset. In addition, in three entries, the performance of our model has improved by about 1% compared to second place. This shows that in different categories, the model can understand the complex relationships between question–image pairs. The results indicate that adversarial learning with a bidirectional attention model outperforms the previous published methods on both the test-standard and test-dev datasets using Faster R-CNN and glove.

### 4.5. Qualitative Results

**Figure 3:**
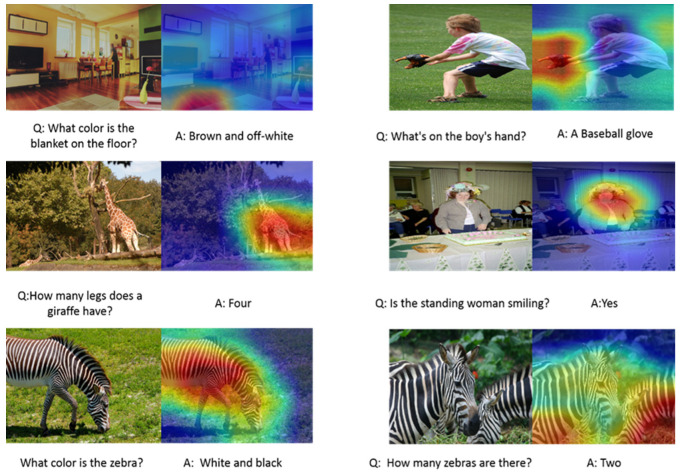
Typical experiment results of related images and questions from the VQA2.0 dataset. The image on the left of each row is related to the question. The image on the right of each row is a visualization of the attention maps related to the question. The text below the image is the question and answer related to the image.

In order to understand the internal mechanism of our model, we visualize the attention maps that ALBA generates for predicting answers. Figure 3 indicates the visualization of the related questions and predicted answers from our model ALBA. The color of attention transitions from red to blue, representing the attention weight value from high to low. The image area most relevant to the problem is highlighted in red. The visualization of attention maps indicates that the capability of ALBA is to fuse related and irrelevant image together according to the question to find the correct answers. In addition, we have conducted comprehensive tests on different types of questions. From the results of the four different question types above, it can be seen that the model has good robustness and comprehensive understanding.

**Figure 4:**
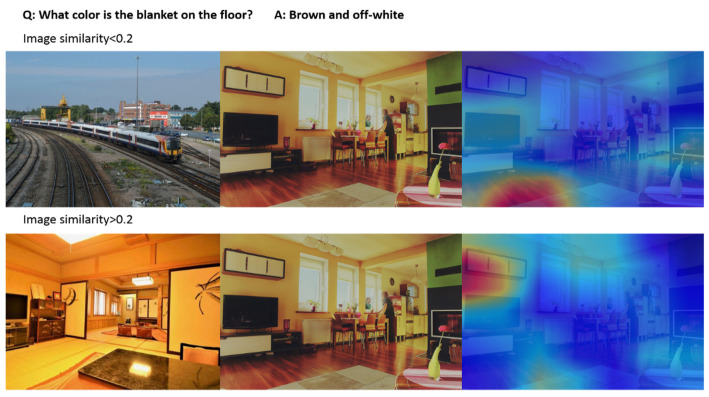
Comparison of the influence of image similarity on attention mechanism.

The similarity between images is also an important factor that affects the attention mechanism. From the above data and analysis, it can be seen that the smaller the similarity, the more accurately the model can improve. It can be seen from Figure 4 that when the image similarity is greater than 0.2, the model cannot answer the question accurately, and the focus is on the wall that is not related to the question. When the image similarity is less than 0.2, the model can effectively integrate the question features and image features, take advantages of the attention mechanism, and focus on the target.

## 5. Conclusions

In this paper, we provide three contributions for solving VQA problems. First, we propose to use adversarial learning methods to use images that are not related to the problem to improve the model’s understanding and reasoning ability.

Second, the bidirectional attention mechanism is used to extract the features of the effective area of the image and then fuse with the question, which reduces the image redundancy and the amount of calculation and makes the attention mechanism of the model more effective.

Third, the VQA accuracy of our model is based on Faster-RCNN and glove, and it outperforms other proposed algorithms. Furthermore, our model has strong adaptability and robustness to different types of questions.

Although our model improves the understanding of the problem and thus the accuracy, it does not have the ability to reason. This will be an optional direction for future model improvement. We aim to explore the logic and spatial reasoning capabilities of the model. Although the paper is not a breakthrough in the field, it can give an alternative direction to solve the VQA problem.

## Figures and Tables

**Table 1:** VQA 2.0 validation score.

	All	Yes/No	Numbers	Other
Reference model	72.12	88.12	53.79	62.38
Image similarity				
Correlation (*p* > 0.2)	71.58	87.1	52.7	61.17
Question embedding				
100-dimensional glove and forward GRU	71.15	87.23	52.69	61.12
200-dimensional glove and forward GRU	70.98	87.01	51.69	62.01
300-dimensional glove and 2-layer forward GRU	71.34	87.54	52.57	61.88
Image features				
ResNet-200 global features (K = 1)	67.12	85.24	51.01	60.98
ResNet-200 features 14 × 14 (K = 196)	68.83	85.84	51.71	61.58
ResNet-200 features downsampled to 7 × 7 (K = 49)	69.59	86.17	51.97	61.93
Attention mechanism				
CNN 7 × 7 features	65.59	84.20	50.75	59.76
ResNet-200 7 × 7 features	66.41	85.68	51.91	60.74
Adversarial learning				
Without adversarial learning	68.78	86.09	52.84	60.62

**Table 2:** Comparison of the results between the proposed method and the other published methods on VQA2.0 using similar settings.

Model	Test-Dev				Test-Standard
	Overall	Yes/No	Number	Other	Overall
VQA team [35]	57.75	80.5	36.77	43.08	58.16
SMem-VQA [14]	57.99	80.87	37.32	43.12	58.24
SAN [12]	58.7	79.3	36.6	46.1	58.9
FDA [36]	59.24	81.14	36.16	45.77	59.54
HQIC [10]	61.8	79.7	38.7	51.7	62.1
FR-VQA	62.43	77.18	33.52	56.09	-
RAU [37]	63.3	81.9	39	53	63.2
DAN [38]	64.3	83	39.1	53.9	64.2
MLB [39]	65.08	84.14	38.21	54.87	65.07
MFB [30]	65.9	84	39.8	56.2	65.8
DCN [40]	66.89	84.61	42.35	57.31	67.02
Count [41]	68.09	83.14	51.62	58.97	68.41
VisualBERT [42]	71.03	87.39	52.64	61.01	71.19
MCAN+VC [43]	71.5	87.09	53.82	61.97	71.72
ALBA (Ours)	72.12	88.12	53.79	62.38	72.33

## Data Availability

Publicly available datasets were analyzed in this study. Available online: https://visualqa.org/download.html (accessed on 26 April 2021).
